# The AdHOC (age, head injury, oxygenation, circulation) score: a simple assessment tool for early assessment of severely injured patients with major fractures

**DOI:** 10.1007/s00068-020-01448-4

**Published:** 2020-07-26

**Authors:** Adrian Knoepfel, Roman Pfeifer, Rolf Lefering, Hans-Christoph Pape, TraumaRegister DGU

**Affiliations:** 1 Department of Trauma, University Hospital Zurich, Rämistrasse 100, 8091 Zurich, Switzerland; 2 Institute for Research in Operative Medicine (IFOM), University Witten/Herdecke, Cologne, Germany

**Keywords:** Mortality prediction, Score, Trauma, Severely injured

## Abstract

**Purpose:**

We sought to develop a simple, effective and accurate assessment tool using well-known prognostic parameters to predict mortality and morbidity in severely injured patients with major fractures at the stage of the trauma bay.

**Methods:**

European Data from the TraumaRegister DGU^®^ were queried for patients aged 16 or older and with an ISS of 9 and higher with major fractures. The development (2012–2015) and validation (2016) groups were separated. The four prognostic aspects Age, Head injury, Oxygenation and Circulation along with parameters were identified as having a relevant impact on the outcome of severely injured patients with major fractures. The performance of the score was analyzed with the area under the receiver operating characteristics curve and compared to other trauma scores.

**Results:**

An increasing AdHOC (Age, Head injury, Oxygenation, Circulation) score value in the 17,827 included patients correlated with increasing mortality (0 points = 0.3%, 1 point = 5.3%, 2 points = 15.6%, 3 points = 42.5% and 4 points = 62.6%). With an AUROC of 0.858 for the development (*n* = 14,047) and 0.877 for the validation (*n* = 3780) group dataset, the score is superior in performance compared to the Injury Severity Score (0.806/0.815).

**Conclusion:**

The AdHOC score appears to be easy and accessible in every emergency room without the requirement of special diagnostic tools or knowledge of the exact injury pattern and can be useful for the planning of further surgical treatment.

## Background

In severely injured patients, early assessment of the patient is crucial for acute care and general management [1, 2]. Existing scoring systems validated for pre-hospital use regularly include parameters of circulation, respiration and head injury [Trauma Score (TS) [3], Revised Trauma Score (RTS) [4], and physiologic trauma score [5]].

In contrast, most scores developed for in-house use require the complete set of diagnoses, including an analysis of lab data of multiple systems. This has been described for the Trauma Score and Injury Severity Score (TRISS) [6], Injury Severity Score (ISS) [7], New Injury Severity Score (NISS) [8], A Severity Characterization of Trauma (ASCOT) [9], and Hospital Trauma Index (HTI) [10]. Moreover, the Trauma Associated Severe Hemorrhage (TASH) score [11] and the Revised Injury Severity Classification (RISC) score [12], which was later revised further to allow for ease of application (RISC II [13]), still require a sustained set of parameters. De Munter et al. reviewed numerous trauma scores established for the prediction of mortality [14]. Most of the published trauma scores are based on the TRISS, Acute Physiology and Chronic Health Evaluation (APACHE) [15] or ASCOT, and represent modifications of these or new combinations with other variables. They postulated that the most accurate trauma score should be developed and validated in a large sample size and using multiple imputation models to address missing values. Other conditions include continuous variables as this leads to more accurate results than categorical or dichotomous variables.

Recently, several groups have developed new scoring systems, like the Emergency Trauma Score (EMTRAS) [16], and the Glasgow Coma Scale, Age, systolic blood Pressure Score (GAP) [17] for the prediction of post-traumatic mortality with higher accuracy (Table 1) than the original pre-hospital scores [3, 4].

**Table 1:** Recent Trauma Scores for Outcome Prediction

Score	Parameters			Scoring
	Demographical	Physiological	Other	
PTS [5] (*n* = 7602)	Age (con)	SIRS (con), GCS (con)		Coefficients
EMTRAS [16] (*n* = 6100)	Age (cat)	GCS (cat), Base excess (cat), Prothrombin time (cat)		0–12
MGAP [27] (*n* = 2363)	Age (dich)	GCS (con), SBP (cat)	Blunt vs. penetrating (dich)	3–29
GAP [17] (*n* = 27,154)	Age (dich)	GCS (con), SBP (cat)		3–24
mREMS [22] (*n* = 429,711)	Age (cat)	SBP (cat), HR (cat), RR (cat), Oxygen Saturation (cat), GCS (cat)		0–26

Clinically used trauma scores for the prediction of mortality with fast and easily available parameters in the early phase of resuscitation (the goal of each score is described in Table 4). Only the number of patients included into the score calculations is shown. The variables are classified as continuous (con), categorical (cat) and dichotomous (dich)

*PTS* Physiologic Trauma Score, *SIRS* Systemic Inflammatory Response Syndrome, *EMTRAS* Emergency Trauma Score, *MGAP* Mechanism, Glasgow Coma Scale, Age and arterial Pressure score, *GAP* Glasgow Coma Scale, Age, systolic blood Pressure score, *mREMS* modified Rapid Emergency Medicine Score, *SIRS* systemic inflammatory response syndrome, *GCS* Glasgow Coma Scale, *SBP* systolic blood pressure, *HR* heart rate, *RR* respiratory rate, *pRBCs* packed red blood cells, *NISS* New Injury Severity Score

Patient prognosis based on laboratory parameters is well known to be relevant, yet it may not be available within a short period of time [18] or needs to be tested sequentially [19]. Likewise, the rapid involvement of computed tomography [20] may not be available, depending on the geographic location or the trauma system involved [21]. Depending on the diagnostic availabilities, the injury severity score may change over time, as the numbers of diagnoses are developing (e.g. hollow viscous injuries, secondary splenic rupture etc.), and may therefore be associated with a lack of accuracy. This is among the reasons to focus on more simplified approaches [22]. Based on these prerequisites, we sought to develop an easier approach whilst maintaining the accuracy of prediction.

Based on a large database with prospectively gathered data, we tested the following hypotheses:

1. A score based on easily and fast available data completed at the end of emergency room treatment can be effective in predicting complications and mortality.
2. The accuracy of this score provides a rapid overview of patient status and a good estimate of threatening complications and can, therefore, help the medical team to plan further surgical treatment.

## Methods

### TraumaRegister DGU^®^

The TraumaRegister DGU^®^ of the German Trauma Society (Deutsche Gesellschaft für Unfallchirurgie, DGU) was founded in 1993. The aim of this multi-center database is the pseudonymized and standardized documentation of severely injured patients. Data are collected prospectively in four consecutive time phases from the time of the accident until discharge from hospital: (A) Pre-hospital phase, (B) Emergency room and initial surgery, (C) Intensive care unit, and (D) Discharge. The documentation includes detailed information on demographics, injury pattern, comorbidities, pre- and in-hospital management, intensive care unit treatment, relevant laboratory findings, including data on transfusion, and patient outcome. The inclusion criteria are admission to hospital via the emergency room with subsequent intensive care unit (ICU)/intensive care medicine (ICM) care or reaching the hospital with vital signs and dying before admission to the ICU.

The infrastructure for documentation, data management, and data analysis is provided by the Academy for Trauma Surgery (AUC—Akademie der Unfallchirurgie GmbH), a company affiliated with the German Trauma Society. The scientific leadership is provided by the Committee on Emergency Medicine, Intensive Care and Trauma Management (Sektion NIS) of the German Trauma Society. The participating hospitals submit their pseudonymized data into a central database via a web-based application. Scientific data analysis is approved according to a peer review procedure established by Sektion NIS.

The participating hospitals are primarily located in Germany (90%) and an increasing number of hospitals in other countries contribute data as well (now from Austria, Belgium, China, Finland, Luxembourg, Slovenia, Switzerland, The Netherlands, and the United Arab Emirates). Currently, approx. 35,000 cases from almost 700 hospitals are entered into the database per year. Participation in the TraumaRegister DGU^®^ is voluntary. For hospitals associated with TraumaNetzwerk DGU^®^, however, the entry of at least a basic data set is obligatory for reasons of quality assurance.

The present study is in line with the publication guidelines of the TraumaRegister DGU^®^ and registered as TR-DGU project ID 2014-036.

### Patients

For data acquisition, the TraumaRegister DGU^®^ was used. Adult trauma cases (age ≥ 16 years) with relevant Trauma (ISS ≥ 9) were included if they had any pelvic, femur or tibia fracture as many multiple trauma are associated with major fractures and multiple minor injuries were not in the focus. These cases were admitted between January 1, 2012 and December 31, 2015 for the development dataset and between January 1, 2016 and December 31, 2016 for the validation data set. Exclusion criteria were secondary admissions and early transfer to another hospital (< 48 h). Patients documented with the basic dataset only were also excluded since no data regarding initial surgery were available.

### Definitions

The Injury Severity Score (ISS) [7] and the New Injury Severity Score (NISS) [8] were used to measure injury severity in the study population. The Glasgow Coma Scale (GCS) was taken to grade unconsciousness [23]. The Eppendorf Cologne Scale (ECS) was used to further assess head injury. The ECS consists of 3 categories, pupil reactivity, pupil size and motor response, and is calculated by summing the values of each subscore to a maximum score of 8; it is more accurate in the prediction of outcome and traumatic brain injury (TBI) than the GCS [24]. For the definition of multiple organ failure (MOF), the Sequential Organ Failure Assessment score (SOFA) was used [25]. MOF was diagnosed in patients with three or more points in a specific organ with at least two organs failing at the same time. The SOFA score was not used to make any outcome predictions. Death during hospital treatment of patients who were admitted to the ICU alive due to a direct or indirect consequence was measured as mortality. RISC II was used to predict outcome in the study populations [13].

### Endpoints

The primary endpoint for the assessment of outcome was in-hospital mortality.

As secondary endpoints, multi-organ failure (MOF), Intensive Care Unit Length of Stay (ICU LOS) and Hospital LOS were selected.

### Identification of prognostic aspects and parameters

After reviewing the literature and previous investigations of the TraumaRegister DGU^®^, four prognostic aspects are known for their impact on the outcome of patients with multiple injuries. Several well-known and easily available parameters are used to assess these aspects. To reach the goal of this study, only parameters which could be evaluated in the emergency room within 30 min of admission were included. One parameter within a prognostic aspect being above or below the defined threshold level means that the whole prognostic aspect is positive so one point can be added to the score total. The threshold levels for the parameters were chosen so that they correlated with a mortality of at least 20%. This value was chosen because it is well above the average mortality of the trauma population of the TraumaRegister DGU^®^ and, therefore, implies a worse outcome than average, which is clinically relevant. Furthermore, they should already be used as cutoffs in everyday clinical setups so that they can be easily remembered. As the purpose of the new scoring system was to be as easy as possible, we did not weigh the prognostic aspects differently, which led to a score with a minimum total of 0 and a maximum total of 4. The four identified prognostic aspects are:

1. “Age”, including the patient age at admission.
2. “Head”, including GCS, Eppendorf–Cologne Scale (ECS) motor function, ECS pupil size and ECS pupil reactivity.
3. “Oxygenation”, including base excess (BE), presence of hemothorax and Horowitz ratio (partial pressure of arterial oxygen (PaO_2_) [mmHg]/fraction of inspire oxygen [FiO_2_]) in intubated patients.
4. “Circulation”, including Hemoglobin (Hb), International Normalized Ratio (INR), transfusion of packed red blood cells (pRBC), and shock, defined as admission systolic blood pressure (SBP) ≤ 90 mmHg.

“Age” has been proven to be a relevant predictor for the outcome of severely injured patients in multiple investigations [9, 13, 22, 26, 27]. We did not use associated parameters, such as the American Society of Anesthesiologists (ASA) (classification of physical status) [28], because it is only of moderate accuracy and also only of moderate inter-observer reliability [29]. The medical history was not used because it is often not available in the trauma bay. We think that age is a good indicator for comorbidities and that those comorbidities may be the reason why elderly patients have a worse outcome than younger individuals with comparable injuries.

For “Head injuries”, both the GCS [23] and the Eppendorf–Cologne Scale (ECS) [24] were used. The latter is more accurate in the prediction of the presence of traumatic brain injury and is able to differentiate the most critical GCS 3 patients better than the GCS itself [24, 30]. It consists of the parameters motor function, pupil size and pupil reactivity, which were implemented in the prognostic aspect head injury and dichotomized.

Within “Oxygenation”, we took BE [31, 32] as a physiologic parameter, as it is an indicator of acidosis, one of three problems in the triad of death in trauma [33]. Hemothorax, as an easily available radiographic or even sonographic parameter, could be identified to have a relevant impact on the survival of severely injured patients in our study population. As the last parameter and indicator for acute lung injury [34], we used the Horowitz ratio (PaO_2_ [mmHg]/FiO_2_) in intubated patients [35–37]. Although the Horowitz ratio is only recorded in the TraumaRegister DGU^®^ if the patient is intubated and mechanically ventilated, the parameter is still useful due to the high intubation rate in the recorded population [38].

For the prognostic aspect “Circulation”, shock [3, 26, 39], low hemoglobin (Hb) [11, 37], transfusion of pRBC [40], and high INR as a sign of coagulopathy and, therefore, also one of the three problems in the triad of death in trauma [13, 33, 40, 41] were identified. These parameters have been proven to have a significant impact on the outcome in severely injured patients. In modern trauma centers, several rapid tests other than the INR can be used to assess whether the patient has a coagulopathy [42] and they could be implemented into routine assessment of coagulopathy in trauma patients.

**Fig. 1:**
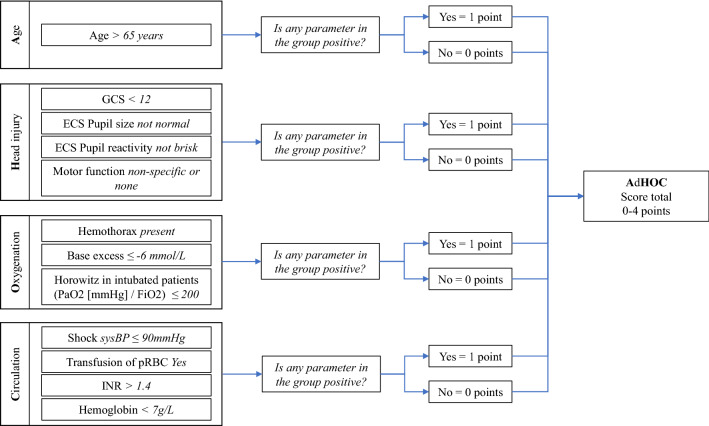
The AdHOC Score. *GCS* Glasgow Coma Scale (27), *ECS* Eppendorf–Cologne Scale (28), *SBP* admission systolic blood pressure, *pRBC* packed red blood cells, *INR* international normalized ratio

The first letters of the prognostic aspects (age, head, oxygenation, circulation) were used to name the AdHOC Score. The score is illustrated in Fig. 1.

### Statistical analysis

Continuous values are presented as mean plus standard deviation (± SD) and median (MD), whereas incidences are shown as percentages. The odds ratios (OR) with 95% confidence intervals were calculated using multivariate logistic regression analysis. The predictive power of the score was analyzed by the area under the ROC curve (AUROC) with 95% confidence intervals. Data were analyzed using the Statistical Package for the Social Sciences (SPSS version 24, IBM Inc., Armonk, USA).

## Results

### Demographic data

The inclusion criteria were met by 14,047 patients for the development group. Their mean injury severity score was 23.8 points, mean NISS was 27.6 and the predicted mortality calculated with the RISC II was 12.2%. Overall, 66.5% of patients were male and 97.5% were victims of blunt trauma. The mean time to death was 7.6 days. Within the entire population, 43% (*n* = 6075) had femur fractures, 34% (*n* = 4715) had tibia fractures and 49% (*n* = 6904) had pelvic ring injuries. The overall in-hospital mortality was 11.9%, the mean ICU LOS was 9.9 days, and the in-hospital LOS was 24.5 days. Overall, 26.0% (*n* = 3652) of patients developed a MOF. The demographic data for the development and validation group are shown in Table 2. The mortality and correlating odds ratios for prognostic aspects as well as the mortality rates correlating with the parameters within prognostic aspects are displayed in Table 3. The mortality of the prognostic aspects “head”, “oxygenation” and “circulation” is lower than the parameters within due to overlapping positive parameters in patients who died; there is less overlap of different parameters from a prognostic aspect in patients who survived.

**Table 2:** Patient characteristics

	Development data set (2012–2015)	Validation data set (2016)
Number of patients	14,047	3780
Mean age [mean/median (SD)]	49.9/49 (21.4)	48.8/49 (20.7)
Male patient	66.5%	68.7%
Blunt trauma	97.5%	98.3%
ISS [mean/median (SD)]	23.8/20 (13.9)	24.7/22 (13.8)
NISS [mean/median (SD)]	27.6/24 (15.0)	28.7/27 (15.0)
RISC II	12.2%	11.8%
GCS < 12 (%)	24.1%	24.3%
GCS [mean/median (SD)]	12.4/15 (4.1)	12.4/15 (4.1)
ECS [mean/median (SD)]	1.1/0 (1.9)	1.0/0 (1.8)
AIS Head ≥ 4	13.5%	15.4%
Prehospital CPR	2.9%	3.3%
SBP ≤ 90 mmHg	15.5%	14.7%
INR > 1.4	15.6%	11.2%
Hb < 7 g/L	3.8%	2.8%
Hemothorax	10.0%	10.3%
BE ≤ -6 mmol/L	22.3%	17.9%
Horowitz ratio (PaO_2_ [mmHg]/FiO_2_) ≤ 200 in intubated Patients	52.5%	46.4%
MOF	26.0%	24.5%
ICU LOS [mean/median (SD)]	9.9/5 (13.0)	8.5/3 (12.3)
In Hospital LOS [mean/median (SD)]	24.5/19 (21.6)	24.1/19 (20.9)
Mortality	11.9%	11.5%
Days to death [mean/median (SD)]	7.6/2 (13.8)	8.4/2 (14.1)

Demographic data of the development and validation data set

*SD* standard deviation, *ISS* Injury Severity Score [7], *NISS* New Injury Severity Score [8], *RISC II* Revised Injury Severity Classification II [13], *GCS* Glasgow Coma Scale [23], *ECS* Eppendorf–Cologne Scale [24], *AIS* abbreviated injury scale, *CPR* cardiopulmonary resuscitation, *SBP* admission systolic blood pressure, *INR* international normalized ratio, *Hb* hemoglobin, *BE* base excess, *PaO*_*2*_ partial pressure of arterial oxygen, *FiO*_*2*_ fraction of inspired oxygen, *MOF* multiple organ failure [25], *ICU* intensive care unit; LOS, length of stay

**Table 3:** Prognostic aspects and parameters

Prognostic aspect	Parameter	Threshold	Mortality (%)	Adjusted OR	95% confidence interval for OR
Age			21.8	4.9	4.3–5.5
	Age	> 65	21.8		
Head			27.7	5.1	4.5–5.8
	GCS	< 12	33.5		
	ECS motor function	Non-specific or none	43.2		
	ECS pupil size	Not normal	40.1		
	ECS pupil reactivity	Not normal	35.6		
Oxygenation			27.4	2.6	2.3–3.0
	Hemothorax	Present	28.4		
	Base excess	≤ − 6 mmol/L	34.6		
	Horowitz ratio (PaO_2_ [mmHg]/FiO_2_) in intubated patients	≤ 200	28.5		
Circulation			26.5	4.1	3.6–4.7
	Shock	SBP ≤ 90 mmHg	33.7		
	Transfusion of RBC	Yes	27.7		
	INR	> 1.4	26.6		
	Hemoglobin	< 7 g/L	48.8		

The four prognostic aspects and the associated threshold levels of the parameters with the corresponding mortality rates and adjusted OR for the prognostic aspects in the development group data set

*GCS* Glasgow Coma Scale [23], *ECS* Eppendorf–Cologne Scale [24], *PaO*_*2*_ partial pressure of arterial oxygen, *FiO*_*2*_ fraction of inspired oxygen, *SBP* admission systolic blood pressure, *pRBC* packed red blood cells, *INR* international normalized ratio

### Mortality and secondary endpoints associated with score value

**Fig. 2:**
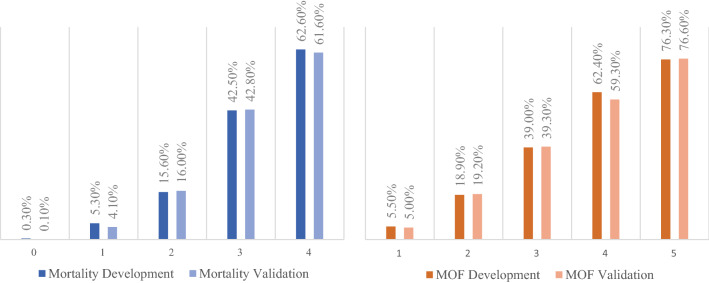
Correlation between AdHOC Score value and mortality (left), multiple organ failure (MOF) (right) in the development and validation sample

Figure 2 shows the association between the value of the AdHOC scoring system and mortality. It demonstrates that zero or one positive prognostic aspect is associated with a quite low mortality (0.3% and 5.3%) and that there is a sharp increase in the mortality rate (at least 15.6%) if two or more prognostic aspects are positive.

Furthermore, an increasing AdHOC Score value in the development group is associated with an increasing ICU LOS from 0 to 2 positive prognostic aspects (0 = 4.1 days, 1 = 7.2 days, 2 = 13.2 days) and then plateaued for 3 (14.3 days) and 4 (12.1 days) positive prognostic aspects. For the Hospital LOS, we found that the LOS increased for from 0 to 2 positive prognostic aspects (0 = 21.2 days, 1 = 24.8 days, 2 = 29.6 days), but thereafter were able to see a decrease in Hospital LOS with 3 (25.6 days) and 4 (18.1 days) positive prognostic aspects. The findings were similar for the validation group.

### Validation group

In comparison of the data from 2012 to 2015 versus those of 2016, we found comparable ISS and NISS values and similar mortality rates. Overall, the data of the two cohorts were mainly the same and, therefore, comparable (Table 2).

### ROC results

Table 4 shows different frequently used scores in trauma and their area under the receiver operating characteristic curve (AUROC). RISC II showed the highest AUROC in both data sets (development data set: 0.929, validation data set: 0.942). The modified rapid emergency medical score (mREMS) [22], using age, physiology and clinical findings, showed better values in the development and validation group than the older trauma-related scores like ISS and NISS. The AdHOC has an AUROC of 0.858 in the development data set and 0.877 in the validation data set, which is comparable to the mREMS and higher than ISS and NISS.

**Table 4:** AUROC comparison

Score	Development dataset (2012–2015)	Validation dataset (2016)	Time of calculation/use				Goal of the score
			Pre-hospital	Trauma bay (30 min)	In-Hospital (hours-days)	Quality control/comparison	
AdHOC score	0.858 [0.849–0.866]	0.877 [0.862–0.892]		x			Easy and early prediction of in-hospital mortality and complications
RISC II [13]	0.929 [0.923–0.935]	0.942 [0.931–0.953]			x	x	Outcome prediction and comparison between different trauma centers and trauma populations
ISS [7]	0.806 [0.794–0.819]	0.815 [0.792–0.838]			x	x	Assessment of injury severity and mortality prediction
NISS [8]	0.813 [0.800–0.825]	0.824 [0.802–0.847]			x	x	Assessment of injury severity and mortality prediction
TRISS [26]	0.893 [0.882–0.904]	0.905 [0.886–0.924]			x	x	Assessment of injury severity and mortality prediction, outcome comparison
RTS [4]	0.792 [0.733–0.811]	0.800 [0.765–0.835]	x				Pre-hospital mortality prediction
GCS [23]	0.788 [0.774–0.802]	0.805 [0.780–0.830]	x	x	x	x	Early prediction of traumatic brain injury, length of coma and mortality
mREMS [22]	0.864 [0.855–0.873]	0.879 [0.862–0.896]	x	x			Easy pre-hospital and emergency department in-hospital Mortality prediction
GAP [17]	0.858 [0.850–0.867]	0.859 [0.842–0.876]		x			Easy and early prediction of in-hospital mortality
PTS [5]	0.868 [0.858–0.877]	0.876 [0.859–0.894]		x			Easy and early prediction of in-hospital mortality
PTGS [40]	0.842 [0.831–0.853]	0.854 [0.833–0.875]		x			Easy discrimination of polytrauma patients at risk for complications on admission

The area under the receiver operating characteristics curve (AUROC), with 95% confidence interval, calculated for several trauma scores

*RISC II* Revised Injury Severity Classification II, *ISS* Injury Severity Score, *NISS* New Injury Severity Score, *TRISS* Trauma an Injury Severity Score, *RTS* Revised Trauma Score, *GCS* Glasgow Coma Scale, *mREMS* modified Rapid Emergency Medicine Score, *GAP* Glasgow Coma Scale, Age, and Systolic Blood Pressure score, *PTS* Physiologic Trauma Score, *PTGS* PolyTrauma Grading Score

## Discussion

Trauma-related deaths represent the major cause of deaths worldwide, with road injuries being the 5th most frequent cause of Years of Life Lost (YLL) (1.34 million deaths and 61.4 million YLL) [43]. It is well known that the rapid identification of severely injured patients and adequate referral to a Level I trauma center influences mortality, complications and LOS [44]. Furthermore, the identification of at risk patients as they arrive at the trauma bay is of utmost importance for further treatment plans, the outcome and to minimize complications [1, 2]. Current scoring systems have been criticized as they were thought to be complex [13, 26]. Less complex scores are discussed as they lack accuracy in outcome predictions or may be too difficult to calculate rapidly and easily.

Our main results are the following:

1. We were able to identify four crucial prognostic aspects with parameters (age, head injury, oxygenation, and circulation) for outcome prediction in trauma patients.
2. The score is currently at a proof of concept status and might be easier to use in the trauma bay than other trauma-related scores.
3. The predictive accuracy for mortality is superior to most other previously developed trauma scores designed for the trauma bay.
4. The score may be used for decision making in the trauma bay.

### Feasibility

The AdHOC score is more applicable in the trauma bay to assess severely injured patients than most other scores for several reasons. The parameters are available early in a trauma bay and are objective, the parameters are reliable and missed injuries do not play a role; this is in contrast to scores using the AIS [45], ISS or NISS [46]. With the exception of the RISC II [13], all trauma scores require a full set of parameters to be calculated. In the AdHOC score, having all parameters within a prognostic aspect is not necessary and the score can be calculated without all the findings. In particular, if one of the parameters within a prognostic aspect has met the threshold level, the prognostic aspect already adds a point to the score total and the other parameters within this prognostic aspect are no longer of interest for the score calculation. The calculation itself is very easy due to the dichotomous variables used and the maximum total score being 4 points only. Weighing the prognostic aspects correlating to their odds ratios (Table 3) would have led to a more complicated calculation of the score. We, therefore, accepted slightly different odds ratios for the prognostic aspects to maintain the simplicity of the score. All other scores have much higher point total due to the use of categorized or continuous variables, and their calculation becomes even more difficult due to the different values given to those variables. Some trauma scores use coefficients correlated to certain values of those categorized or continuous variables, which makes the calculation even more difficult.

Moreover, the parameters used are obtainable in countries where the western society emergency room conditions may not be available and the AdHOC score, therefore, can be of value in the assessment of severely injured patients, especially in those countries. If slightly modified and after further evaluation the score could possibly even be used for preclinical assessment.

De Munter et al. proposed that continuous variables should not be categorized or dichotomized for the better discrimination of patient outcomes [14]. Setting thresholds for the parameters used and dichotomizing those variables make the score less discriminating than using categories or even continuous variables. In terms of quality control and best comparability between different trauma centers, this is true. However, it is not a necessity in the acute evaluation and treatment of severely injured patients in a trauma bay. The score should enable the treating physicians to obtain a rapid assessment of the patients’ condition.

### Accuracy

Some may argue that not using injury patterns for a trauma scoring system makes this score imprecise [14] and should probably not be used to assess patients in the emergency room or a trauma bay. However, many previous studies have already shown that a scoring system using only clinical findings, lab results and the physiological state of a patient leads to an accurate score that is superior to AIS-dependent scoring systems like the ISS or the NISS [22, 27]. Scores like the TRISS [26] and the RISC II [13] are clearly more accurate, but require further radiologic procedures to be completed like the ISS or the NISS [7, 8]. Because we intended to create an easy score that could even be used in less developed countries, the AdHOC should not be compared to these highly demanding scores. We also think that not being dependent on the exact knowledge of the injury pattern and, therefore, the AIS and ISS are strengths of the AdHOC, because there are several limitations of the AIS, as shown by Ringdal et al. [47].

### Use of the score

With the score, we can predict the general outcome of severely injured patients with major fractures at a trauma center and help the physicians with decision making. We believe that severely injured patients with an AdHOC score of 0 (mortality 0.3%, MOF 5.5%) or 1 (mortality 5.3%, MOF 18.9%) can be considered stable, whereas patients with a score of 3 (mortality 42.5%, MOF 62.4%) or 4 (mortality 62.6%, MOF 76.3%) have a poor outcome and, therefore, must be stated as unstable. Patients with a score of 2 points (mortality 14.4%, MOF 37.7%) are at an intermediate risk (“borderline patients”) [2]. It is not possible to advise a certain treatment plan for patients in different outcome groups due to the study design. Further investigation has to be performed to determine whether a certain group benefits from a special treatment plan.

Patients with two points and their response to resuscitation should be further evaluated, because they can improve or deteriorate quickly. In particular, “Circulation” and “Oxygenation” can be easily improved within minutes after the arrival of a patient at a trauma bay, probably leading to a stable patient (e.g. through insertion of a chest tube in tension pneumothorax). We believe that this is also the main reason why those two prognostic aspects have smaller odds ratios than “Age” and “Head”.

### Strengths and limitations

Our study has both strengths and limitations. A strength of this study is the size of the registry (*n* = 14,047) and the use of a validation data group (*n* = 3780) [14]. The data contributed to the database from many different clinics in several countries are of a high quality and prospectively gathered. It is also very important that the score is validated in the same database, unlike other newly published scores, such as the mREMS [22].

The AdHOC score is developed and validated only for patients treated in developed trauma system environments. Different findings may occur outside Europe and less developed environments. Although the AdHOC score was developed and validated for patients with major fractures of the pelvis, femur and tibia, we believe that it can be applied to assess any severely injured patient in the trauma bay because the demographic data do not differ greatly from the total trauma population of the TraumaRegister DGU^®^. Brockamp et al. were able to show that a trauma score for pediatric trauma performed well in the prediction of mortality in adult trauma [41], so the AdHOC score would probably be viable for the assessment of severely injured children. Consequently, the score should be validated in a trauma population outside Europe to prove its worldwide feasibility and those points mentioned above, preferably in a large prospective database, such as the TraumaRegister DGU^®^.

A limitation of many trauma scores in general is that they are not able to predict different major complications like the development of ARDS or wound infections in the further course of the hospitalization [14]. Further investigations should be performed to evaluate the predictive relevance of the score in terms of other major complications or even long-term outcomes.

Furthermore, missing data are a problem in large databases, but are rare in the TraumaRegister DGU^®^. In the case of the AdHOC score, the variables had a high completeness rate above 80%, most of them even > 95% and some even 100% (age, hemothorax). Although a missing variable could alter the score value and underestimate the trauma severity, it is an advantage that several different findings are used to count an aspect as positive. This also compensates for most missing data (e.g. when systolic blood pressure < 90 mmHg is positive a missing hemoglobin value has no further impact). We also checked for each variable whether cases with missing values had similar mortality rates as cases with available data and did not find any relevant difference.

## Conclusion

The score data reconfirm the value of the use of multiple parameters to assess severely injured patients with major fractures and their further treatment. The AdHOC score appears to be easy and accessible in every emergency room without the requirement for special diagnostic tools or knowledge of the exact injury pattern. Further investigations in different trauma systems should be performed to evaluate the value of the score.
